# The effect of cognitive behavioral therapy on stress and anxiety of mothers of girls with precocious puberty symptoms: a randomized controlled trial

**DOI:** 10.1186/s12888-023-05216-7

**Published:** 2023-10-10

**Authors:** Faranak Rahimi, Mojgan Mirghafourvand, Mahmoud Farvareshi, Parisa Yavarikia

**Affiliations:** 1 Department of midwifery, Student Research Committee, Faculty of Nursing and Midwifery, Tabriz University of Medical Sciences, Tabriz, Iran; 2 Social Determinants of Health Research Center, Faculty of Nursing and Midwifery, Tabriz University of Medical Sciences, Tabriz, Iran; 3 Clinical Psychologist, Razi Hospital, Tabriz University of Medical Sciences, Tabriz, Iran; 4 Department of midwifery, Faculty of Nursing and Midwifery, Tabriz University of Medical Sciences, Tabriz, Iran

**Keywords:** Puberty, Precocious puberty, Stress, Anxiety, Quality of life

## Abstract

**Introduction:**

: Precocious puberty in girls has been associated with an increased risk of stress and anxiety in their mothers. This study aimed to investigate the effect of cognitive behavioral therapy (CBT) on perceived stress and anxiety of mothers of girls with precocious puberty symptoms.

**Methods:**

This randomized controlled trial was conducted on 70 mothers of girls with precocious puberty symptoms in Tabriz-Iran, 2021. The participants were randomly assigned to CBT and control groups through blocked randomization. Group counseling was provided to the intervention group in eight sessions of 45–60 min weekly with 5 to 7 women. A booklet containing explanations about puberty was provided for the both groups. Data were collected using the questionnaires of socio-demographic characteristics, Spielberger State-Trait Anxiety Inventory (STAI), Perceived Stress Scale (PSS) and quality of life (SF-36). Independent t-test, ANCOVA, chi-square, and fisher’s exact tests were used to compare the outcomes between the groups.

**Findings:**

: After the intervention, based on ANCOVA test with adjusting the baseline values, mean scores of stress (mean difference (MD): -10.75; 95% confidence interval (95% CI): -11.77 to -9.72; P < 0.001), state anxiety (MD: -14.36; 95% CI: -15.7 to -12.7; P < 0.001) and trait anxiety (MD: -12.8; 95% CI: -14.4 to -11.1; P < 0.001) were significantly lower in CBT group compared to the control group. Also mean score of quality of life (MD: 9.82; 95% CI: -6.74 to -12.90; P < 0.001) was significantly higher in CBT group compared to the control group.

**Conclusion:**

Based on the results, group CBT is effective in reducing stress and anxiety and improving the quality of life of mothers of girls with precocious puberty symptoms. However, more studies are required to make a definite conclusion in this field.

**Trial registration:**

Iranian Registry of Clinical Trials (IRCT): IRCT20110826007418N6. Date of registration: 11/10/2021. URL: https://en.irct.ir/trial/57346; Date of first registration: 11/10/2021.

## Introduction

Puberty is the process of physical maturation when a person becomes capable of reproduction [1]. Adolescence is the most turbulent stage of life, which is a bridge between childhood and adulthood. The World Health Organization (WHO) defines adolescents as those between 10 and 19 years of age. Puberty in girls begins from 10 to 12 years old with the appearance of secondary sexual characteristics [2]. It starts with the accumulation of adipose and results in changes in the bony pelvis and its widening, breast development, and the appearance of dark straight hair on the pubis. The maximum speed of this growth spurt is just before the onset of menstruation [3]. If these physical changes happen before the age of 8, it is called precocious puberty [4].

Risk factors for precocious puberty are improper diet, use of processed foods such as canned goods, obesity, growth hormone therapy [5], gene mutations [6], smoking by the mother during pregnancy [7], and excessive consumption of red meat [8]. A wide range of neurological causes should also be considered, such as central nervous system (CNS) tumors, CNS infections, concussions, hypothyroidism, etc. [9] The prevalence of precocious puberty is about 1:5000 to 1:10000, and it is ten times more common in girls than in boys [10]. In recent research in Iran, it has been reported to be 10.3%; this statistic had a significant association with BMI, frequency of fast food consumption, and birth rank [11]. Precocious puberty in girls is associated with many physical and psychological complications such as an increased risk of early sexual activity, teenage pregnancy, health issues during adolescence, cardiovascular diseases in adulthood, and breast cancer [4]. The physical and emotional changes associated with the early onset of puberty may lead to psychological and social problems. It may affect the quality of life [12]. Precocious puberty is accompanied by stress, anxiety, and depression for the child’s parents.

Studies have shown that most mothers lack sufficient knowledge about puberty [13]. Precocious puberty symptoms in girls may cause unfavorable consequences in their mothers. It leads to mild to severe stress in more than 50% of these mothers and can cause mental and emotional problems [14]. Stress is a reaction against a perceived threat (real or imagined) to an individual’s mental, physical, or spiritual well-being, resulting from a series of physiological responses and adaptations [15]. Occasional anxiety is a normal and unavoidable part of life [16]. It is an unpleasant feeling of worry accompanied by physical symptoms such as palpitation, shortness of breath, sweating, restlessness, and the urge to move. Everyone has experienced the feeling of anxiety [17].

Cognitive-behavioral therapy (CBT) is a combination of cognitive and behavioral approaches. It helps the patient to recognize the distorted thought patterns and ineffective behaviors. Then regular meetings and behavioral interventions are used to change these thoughts and behaviors. The basis of cognitive-behavioral methods is to change the cognitive process [18]. CBT is one of the approaches of psychology, and its goal is to identify and challenge irrational behaviors and thoughts to bring a person to overall mental health. It is one of the short-term treatments with high effectiveness [19].

Previous studies have shown the positive effect of group CBT on occupational stress and anxiety of patients [20, 21]. No studies were found that measured the effect of CBT on stress and anxiety of mothers of girls with precocious puberty symptoms. Precocious puberty in girls causes stress, anxiety, and other psychological problems in their mothers. The present study aimed to determine the effect of cognitive behavioral therapy on stress, anxiety and quality of life of mothers of girls with precocious puberty symptoms.

## Methods

### Study design and participants

It was a randomized controlled trial. Participants were mothers of girls with precocious puberty symptoms, studying in elementary schools in Tabriz-Iran, from July 2021 to October 2021. The sampling was started after approving by the Ethics Committee of Tabriz University of Medical Sciences (IR.TBZMED.REC.1400.227) and registration in IRCT (IRCT20110826007418N6; 11/10/2021).

Inclusion criteria: mothers were required to have a daughter aged 8–9 with precocious puberty symptoms [22], a minimum of secondary school literacy, having a smart phone, having stress and anxiety due to their daughters’ precocious puberty, and a score of 35–65 (moderate and relatively severe) on STAI (State-Trait Anxiety Inventory) and above 21.8 on PSS (Perceived Stress Scale) [23]. Exclusion criteria: having a history of mental disorders according to the person’s statement, experiencing a severe mental health crisis in the last three months, such as the death of a relative, taking neuropsychiatric medications, other physical or mental problems, and participating in similar studies.

Based on the results of the study by Karimi et al. (2018) [24] with considering m_1_ = 108.26 (mean score of anxiety before intervention), and with a default reduction of 20% in anxiety score after intervention (m_2_ = 86.61), SD_1_ = SD_2_ = 20.48, two-sided α = 0.05, power = 95% sample size was calculated as 21 women in each group. The final sample size was 24, considering a drop-out rate of 10%. Based on the perceived stress variable [25] with considering m_1_ = 24.76 (mean score of stress before intervention), and with a default reduction of 20% in stress score after intervention (m_2_ = 19.89), SD_1_ = SD2 = 7.7, α = 0.05, power = 80% sample size was calculated as 32 women in each group. The final sample size was 35, considering a drop-out rate of 10%. The sample size calculated with the stress variable was higher; therefore, the final sample size was 35 women in each group.

### Sampling

The researcher was referred to 35 elementary girl schools in Tabriz, randomly selected from five educational districts. She received the numbers of the mothers from the school officials, then contacted all of them and asked about the appearance of puberty symptoms in their daughters. They were assessed based on the inclusion and exclusion criteria. Potential mothers were invited to attend a face-to-face meeting held at the school. The objectives and methods were fully explained at the meeting, and a written consent form was signed by the mothers who were willing to participate in the study. The questionnaires of Spielberger State-Trait Anxiety Inventory (STAI) and Perceived Stress Scale (PSS) were filled out. Mothers who scored 35–65 (moderate and relatively severe) on STAI and above 21.8 on PSS were included. Next the questionnaires of socio-demographic characteristics and quality of life (SF-36) were completed.

#### Randomization

Participants were randomly assigned to CBT and control groups through blocked randomization, with 4 and 6 block sizes and a 1:1 allocation ratio. A non-involved person performed the random allocation. Sealed opaque envelopes were used for allocation concealment. The envelopes were revealed sequentially as the participants entered the study.

### Intervention

CBT was provided in eight sessions of 45–60 min weekly with 5 to 7 women. The first session was held face-to-face in a quiet and intimate room to introduce and explain the sessions to the mothers. All health protocols and social distancing guidelines were observed. The next sessions were six face-to-face meetings and two virtual meetings through WhatsApp. The meetings were held weekly due to the time limit of schools during the summer break and the spread of Coronavirus. A booklet containing explanations about puberty was provided for the both groups.

The first author (a master’s student in counseling in midwifery) conducted the sessions under the supervision of a Ph.D. in clinical psychology who designed the intervention method. The consultant reminded the participants of the meeting time by calling them the day before each meeting. If a person could not participate at a session, she was invited to another time in the same week. All 35 members of the intervention group completed all the counseling sessions. In addition, counseling was conducted in the vernacular language. The content of the meetings is described in Table 1.

**Table 1:** Content of group cognitive behavioral therapy sessions for mothers who have girls with precocious puberty symptoms

Sessions	Content
1	Explaining the frequency and duration of each session, rules, identification of the problem, the introduction of cognitive behavioral therapy and its components, problem statement, effects of stress, goals, and feedback.
2	mood assessment^1^, the introduction of progressive muscle relaxation and its practice, providing homework: progressive muscle relaxation^2^ twice a day, receiving feedback
3	Review the cognitive-behavioral pattern according to the problem, mood assessment, asking the members to explain their worries, introduction of imagery practice^3^, providing homework: progressive muscle relaxation, and imagery practice.
4	Mood assessment, review of the imagery practice, introduction of the first three columns of the thought record^4^ sheet (situation – automatic thoughts - emotions and mood) and hot thoughts, practicing the sheet using one of the events of last week, providing homework: imagery practice and the first three columns of the thought record sheet, receiving feedback.
5	Mood assessment, discussing the treatment process and its completion, review of the homework, introducing cognitive distortions, completing the three columns of thought record sheet (situation – automatic thoughts - emotions and mood), identification and challenging hot thoughts, providing homework: relaxation technique and completing the three columns of thought record sheet, receiving feedback.
6	Mood assessment, review of the homework, introducing thought challenging^5^ and the seven-column thought record sheet (situation – automatic thoughts - emotions and mood - confirming evidence - rejecting evidence - alternative thinking - re-evaluating), completing the columns during the session, introducing the concept of challenging hot thoughts^6^, providing homework: completing the seven-column thought record sheet and relaxation techniques, receiving feedback.
7	Mood assessment, review of the homework, completion of the seven-column thought record sheet, providing homework: completing the seven-column thought record sheet and relaxation techniques, receiving feedback.
8	Mood assessment, review of the homework and treatment process (CBT techniques), prevention, introducing self-management sessions, providing homework: self-management session, and determining the exact date of the post-test.

^1^ Mood assessment; the participants were asked to rate their mood on VAS scale

^2^ Progressive muscle relaxation involves tensing then relaxing your muscles, one by one

^3^ A mental simulation process that involves the systematic use of imagery to covertly rehearse a movement without actually executing it

^4^ Thought records are tools that help capture, evaluate, and restructure negative automatic thoughts

^5^ Thought challenging practice can help us broaden our focus and include the bigger picture; it reduces anxiety

^6^ Hot thoughts are classed as instant negative reactions to perceived threats or problems

### Data collection tools

Socio-demographics characteristics questionnaire, STAI, PSS-14, and SF36 were used before and four weeks after the intervention.

### Socio-demographics characteristics questionnaire

It included questions about the age of the mother and her daughter, the school grade, the level of education and occupation of the parents, income sufficiency, life satisfaction, and living conditions.

### Perceived stress scale (PSS)

PSS was developed in 1983 by Cohen et al. It has three versions of 4, 10, and 14 that are used to determine general perceived stress over the past month and measure thoughts, feelings, stressful events, and coping methods. This scale also assesses the risk factors of behavioral disorders and indicates the process of stressful relationships. In this research, a 14-item version was used. The scoring is based on the 5-point Likert scale. The minimum and maximum scores are 0 and 56, respectively. The cut-off point is 21.8; a higher score on this scale indicates more stress. Internal consistency and reliability coefficients were obtained through Cronbach’s alpha in the range of 0.84–0.86 in two groups of students and a group of smokers in the smoking cessation program [26]. The reliability of the Persian version has been confirmed by Bastani et al. using the internal consistency method and Cronbach’s alpha of 0.74 [27]. Mohammadi Yeganeh and colleagues have also confirmed the reliability of this tool [28].

#### Spielberger state-trait anxiety inventory (STAI)

This questionnaire was developed by Spielberger et al. (1970) and revised in 1980. The Spielberger anxiety inventory, also known as STAI, includes separate self-measurement scales to measure state anxiety (STAI-y1) and trait anxiety (STAI-y2). This questionnaire has 40 items. Items 1 to 20 include state anxiety with four options (very low, low, high, very high) and items 21 to 40 trait anxiety with four options (rarely, sometimes, often, almost always). This scale has no time limitations. Each STAI item is valued from 1 to 4 based on the answer provided. Higher scores indicate more anxiety symptoms. The reliability coefficient for items 1 to 20 was 0.889; it was 0.864 for items 21 to 40. Reliability in the state and trait anxiety scale based on Cronbach’s alpha was 0.91 and 0.90, respectively [29].

### SF-36 quality of life

36-item Short Form Health Survey (SF-36) was designed by Ware et al. in 1992 in the United States. Its validity and reliability have been investigated in different groups of studies. SF-36 consists of 36 items that evaluate eight health concepts: general health (5 items), physical functioning (10 items), energy/vitality (4 items) and emotional role limitations (3 items), bodily pain (2 items), social functioning (2 items), physical role limitations (4 items), and mental health (5 items). The minimum and maximum scores are 0 and 100, respectively [30]. The reliability and validity of the Persian version were confirmed by Montazeri et al. in Iran (r = 0.7–0.9) [31].

### Visual analog scale (VAS)

A mood assessment occurred at the beginning of each session and it was an opportunity for the therapist to check on how the patient’s mood has been since the last session. Visual analogue scales (VAS) can be used for subjective ratings of mood, emotion, or distress. The participants simply rate the intensity of the sensation on a scale from 0 to 10. It has been found to have good psychometric properties in Iran [32].

### Statistical analysis

Statistical analysis was performed using SPSS version 26. The normality of the quantitative data was assessed using the Kolmogorov-Smirnov (K-S) test, all of which were in the normal range. Independent t tests, chi-square, chi-square by trend and Fisher’s exact tests were used to assess the homogeneity of the study groups. To compare the groups in terms of mean scores of perceived stress and anxiety (primary outcomes) and quality of life (secondary outcome), independent t-test was performed before the intervention and ANCOVA test after the intervention by adjusting baseline values. All analyses were performed based on Intention-to-Treat. P < 0.05 was considered significant.

## Results

**Fig. 1:**
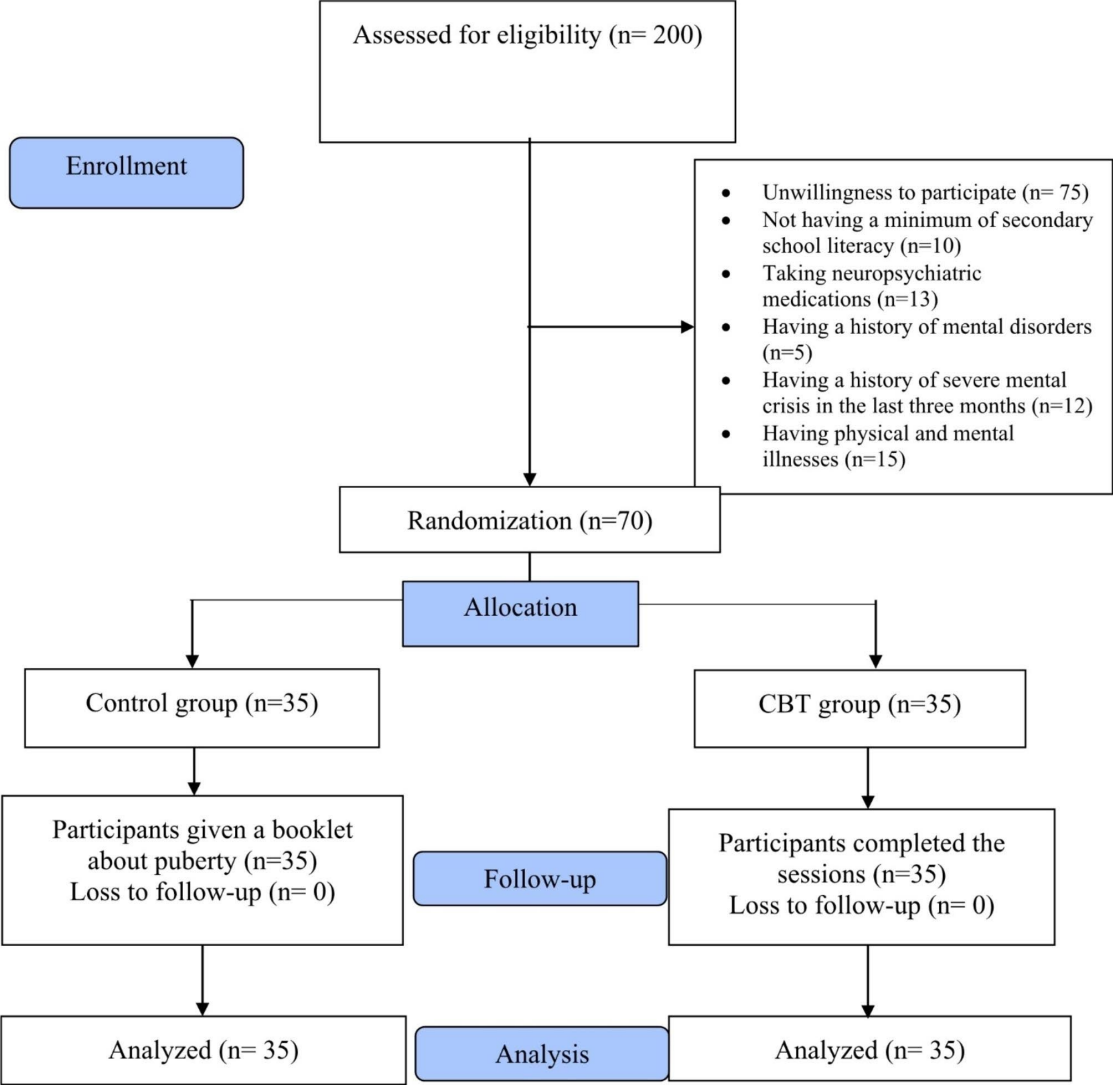
Flow chart of the study

Among the 35 mothers assigned to the counseling group, all of them participated in 8 counseling sessions; there was no dropout from the study (Fig. 1). The socio-demographic characteristics of the participants are shown in Table 2. There was no statistically significant difference between the groups in terms of socio-demographic characteristics except for the girl age, school grade, and father’s education, which was adjusted using ANCOVA.

**Table 2:** Socio-demographic characteristics of mothers in the CBT and control group

Characteristic	CBT^*^ (n = 35)	Control (n = 35)	P-value
	Mean (SD)	Mean (SD)	
**Mother age** (Year)	36.4 (5.06)	34.8 (6.37)	0.269^†^
	**Number (Percent)**	**Number (Percent)**	
**Girl age (Year)**			0.055^§^
8 years	12 (34.3)	21 (60.0)	
9 years	23 (65.7)	14 (40.0)	
**Birth order**			0.326^§^
First-born	24 (68.6)	22 (62.9)	
Second-born	11 (4.3)	3 (28.6)	
Third-born	0 (0.0)	3 (8.6)	
**School grade**			0.001^‡^
Second grade	7 (20.0)	21 (60.0)	
Third grade	28 (80.0)	14 (40.0)	
**Mother’s education**			0.639^‡^
Intermediate	6 (17.1)	7 (20.00)	
High school	4 (11.4)	4 (11.4)	
Diploma	15 (42.9)	10 (28.6)	
University	10 (28.6)	14 (40.0)	
**Mother’s job**			0.367^¥^
House wife	31 (88.6)	29 (82.9)	
Employed	4 (11.4)	6 (17.1)	
**Father’s education**			< 0.001^‡^
Elementary	3 (8.6)	0 (0.0)	
Intermediate	10 (28.6)	2 (5.7)	
High school	9 (25.7)	2 (5.7)	
Diploma	1 (2.9)	20 (57.1)	
University	23 (34.3)	11 (31.4)	
**Father job**			0.617^¥^
Unemployed	1 (2.9)	2 (5.7)	
Employee	7 (20.0)	7 (20.0)	
Worker	10 (28.6)	7 (20.0)	
shopkeeper	6 (17.1)	3 (8.6)	
Other	11 (31.4)	16 (45.7)	
**Income sufficiency**			0.458^‡^
Sufficient	7 (20.0)	8 (22.9)	
Not sufficient	8 (22.9)	11 (31.4)	
Somewhat sufficient	20 (57.1)	16 (45.7)	
**Living place**			0.112^¥^
Private house	23 (76.5)	19 (54.3)	
Rental house	4 (11.4)	9 (25.7)	
Mother’s parents’ house	1 (2.9)	3 (8.6)	
Father’s parents’ house	3 (8.6)	4 (11.4)	
Other	4 (11.4)	0 (0.0)	
**Life satisfaction**			0.124^‡^
Relatively satisfied	25 (71.4)	30 (85.7)	
Dissatisfied	5 (14.3)	4 (11.4)	
Satisfied	5 (14.3)	1 (2.9)	

^*^ Cognitive Behavioral Therapy; ^†^Independent t-test; ^§^Chi-square test; ^¥^ Fisher’s exact test; ^‡^ Liner by liner association

According to the independent t-test, there was no difference in mean stress score between the groups before the intervention. Through the ANCOVA test and adjusting the baseline scores, it was significantly lower in the CBT group after the intervention. There was no difference in mean scores of state and trait anxiety between the groups before the intervention; they were significantly lower in the CBT group after the intervention. (Table 3).

**Table 3:** Comparison of the mean score of mothers’ stress and anxiety between the CBT and control group

Variable	CBT^*^ (n = 35) Mean (SD^†^)	Control (n = 35) Mean (SD^†^)	Mean Difference (95% Confidence Interval)	P-value
**Perceived stress** (Score range: 0 to 56)				
Before intervention	26.5 (4.1)	28.6 (3.0)	2.0 (0.3 to 3.8)	0.198^‡^
4 weeks after intervention	16.6 (1.3)	31.4 (2.2)	-10.7 (-11.7 to -9.7)	< 0.001^§^
**State anxiety** (Score range: 20 to 80)				
Before intervention	44.6 (6.6)	45.7 (3.8)	1.1 (-1.4 to 3.7)	0.385^‡^
4 weeks after intervention	34.2 (3.0)	48.7 (3.1)	-14.3 (-15.9 to -12.7)	< 0.001^§^
**Trait anxiety** (Score range: 20 to 80)				
Before intervention	43.4 (5.2)	44.5 (3.3)	1.0 (-1.0 to 3.1)	0.310^‡^
4 weeks after intervention	34.1 (2.5)	47.0 (3.2)	-12.8 (-14.4 to -11.1)	< 0.001^§^

^*^Cognitive Behavioral Therapy; ^†^ Standard Deviation; ^‡^Independent t-test; ^§^ ANCOVA with adjusting the baseline value and the variables of girl age, school grade, and father’s education: The effect of girl age (P values of PSS:0.530, S-anxiety:0.716, T-anxiety:0.359), school grade (PSS:0.840, S-anxiety:0.969, T-anxiety:0.475), and father’s education (PSS:0.408, S-anxiety:0.996, T-anxiety:0.057) on the results were insignificant

According to the ANCOVA and adjusting the baseline scores, the mean quality of life score in the CBT group was significantly more than the control one after the intervention (Table 4).

**Table 4:** Comparison of the mean score of mothers’ quality of life between the CBT and control group

Variable	CBT^*^ (n = 35) Mean (SD^†^)	Control (n = 35) Mean (SD^†^)	Mean Difference (95% Confidence Interval)	P-value
**Total quality of life** (Score range: 0 to 100)				
Before intervention	40.0 (6.6)	43.5 (5.0)	3.4 (0.6 to 6.3)	0.017^‡^
4 weeks after intervention	51.8 (6.1)	43.8 (4.7)	9.8 (6.7 to 12.9)	< 0.001^§^
**Physical functioning** (Score range: 0 to 100)				
Before intervention	51.5 (14.5)	53.2 (15.6)	1.7 (-5.4 to 8.9)	0.637^‡^
4 weeks after intervention	14.8 (8.4)	58.0 (12.6)	-37.6 (-33.0 to -42.1)	< 0.001^§^
**Physical role limitations** (Score range: 0 to 100)				
Before intervention	57.1 (14.3)	53.5 (15.0)	-3.5 (-10.5 to 3.4)	0.312^‡^
4 weeks after intervention	70 (17.9)	54.2 (16.5)	8.9 (20.4 to -2.5)	0.126^§^
**Bodily pain** (Score range: 0 to 100)				
Before intervention	24.8 (10.9)	33.3 (12.6)	8.5 (2.8 to 14.1)	0.004^‡^
4 weeks after intervention	44.68 (9.94)	31.22 (11.37)	20.8 (24.1 to 17.5)	< 0.001^§^
**General health** (Score range: 0 to 100)				
Before intervention	36.5 (11.0)	39.7 (8.6)	3.2 (-1.5 to 7.9)	0.181^‡^
4 weeks after intervention	58.6 (9.93)	37.02 (8.91)	22.3 (25.9 to 18.6)	< 0.001^§^
**Energy/Vitality** (Score range: 0 to 100)				
Before intervention	27.2 (12.0)	34.7 (6.2)	7.4 (2.8 to 12.0)	0.002^‡^
4 weeks after intervention	54.5 (6.8)	34.4 (6.6)	21.4 (25.9 to 17.0)	< 0.001^§^
**Social functioning** (Score range: 0 to 100)				
Before intervention	33.3 (15.9)	40.1 (12.3)	6.7 (-0.1 to 13.5)	0.053^‡^
4 weeks after intervention	52.8 (15.9)	40.5 (13.8)	17.0 (24.0 to 9.9)	< 0.001^§^
**Emotional role limitations** (Score range: 0 to 100)				
Before intervention	59.1 (18.4)	54.3 (18.5)	-4.8 (-13.6 to 3.9)	0.279^‡^
4 weeks after intervention	65.9 (12.8)	56.2 (17.9)	9.1 (19.3 to -0.9)	0.076^§^
**Emotional health** (Score range: 0 to 100)				
Before intervention	31.0 (9.2)	39.7 (5.0)	8.6 (5.1 to 12.2)	< 0.001^‡^
4 weeks after intervention	53.9 (10.0)	39.6 (4.9)	17.7 (22.8 to 12.6)	< 0.001^§^
**Physical health** (Score range: 0 to 100)				
Before intervention	39.4 (5.6)	42.8 (5.7)	3.4 (0.7 to 6.1)	0.014^‡^
4 weeks after intervention	48.7 (5.1)	42.9 (5.7)	7.4 (10.2 to 4.6)	< 0.001^§^
**Mental health** (Score range: 0 to 100)				
Before intervention	37.0 (7.9)	41.7 (6.1)	4.6 (0.9 to 8.3)	0.014^‡^
4 weeks after intervention	57.1 (7.8)	41.6 (5.9)	17.6 (21.6 to 13.7)	< 0.001

^*^ Cognitive Behavioral Therapy; ^†^ Standard Deviation; ^‡^Independent t-test; ^§^ ANCOVA with adjusting the baseline value and the variables of girl age (P = 0.438), school grade (P = 0.319), and father’s education (P = 0.770): their effect on the total score of quality of life and its subscales were insignificant

## Discussion

The results showed that CBT was effective in reducing stress and anxiety and promoting the quality of life of mothers of girls with precocious puberty symptoms.

CBT significantly reduced the mean stress score in our study. In the study conducted by Izadi et al. (2015), the stress in mothers of children with autism was measured with the Parenting Stress-Short Form (PSI-SF) questionnaire. CBT was provided in seven sessions of 90 min using relaxation techniques, identification of automatic thoughts, and cognitive distortions. It was significantly effective for stress reduction [33]. Shabani et al. (2022) conducted a group-based cognitive behavioral intervention on the stress levels of women with premature ovarian insufficiency. The meetings were provided in eight sessions of 45–60 min once a week. Using PSS, they concluded that CBT can contribute to a reduction in stress [23]. The results of both studies are in line with the present one. The perception of stress and responding to it are affected by previous experiences, current situations, and learned behaviors [21]. CBT approach suggests new thinking and behavioral techniques, taught to replace mothers’ negative thoughts about themselves, the world, and the future. These techniques are helpful in identifying stressful situations and using appropriate coping strategies [25].

CBT significantly reduced the mean anxiety score in our study. In the study of Koochaki et al. (2017), mothers with preterm children who were diagnosed with anxiety disorders by Beck Anxiety Inventory (BAI) participated in 8 sessions of CBT. The sessions were conducted based on the principles of teaching skills, behaviors, and interactions. The participants were followed up immediately after the intervention and three weeks later. They reported a significant reduction in anxiety scores in both stages [34]. In the study conducted by Majidzadeh et al. (2023), the anxiety of women with polycystic ovary syndrome was measured by STAI, and the intervention group underwent 8 sessions of 60–90 min cognitive-behavioral counseling. The mean anxiety score in the counseling group decreased significantly after the treatment [35]. The results of both studies are consistent with the present one. Cognitive-behavioral methods helps identify negative thoughts and replace them with more objective and rational ones. It promotes mental schemas through behavioral techniques, muscle relaxation training, and objectivity in anxiety management which may reduce the mother’s anxiety level [36].

CBT significantly improved the quality of life score in our study. The study of Isazadegan et al. (2013) aimed to investigate the effect of CBT on the lifestyle and quality of life of patients with hypertension. The results showed that CBT increased the lifestyle score and quality of life of patients in the intervention group [37]. The study of Barzegar et al. aimed to determine the effect of teaching biological, cognitive, and emotional characteristics of puberty to mothers on improving the parent-child relationships of girls with precocious puberty. The results showed that teaching these characteristics had significantly affected positive emotions, role-playing with the mother, and the parent-child relationship [38]. The results of both studies are in line with the present one. It seems that there is a close relationship between a person’s belief, self-confidence, self-concept, and the surrounding world with his capability to deal with different life conditions, quality of life, and satisfaction [39]. CBT can improve a person’s way of thinking and quality of life by using techniques of challenging thoughts and beliefs [40].

The review of studies shows the positive effect of CBT on reducing stress and anxiety and increasing quality of life. When using cognitive behavioral skills in stressful situations, people would realize they can make better decisions to achieve the desired results [41]. Improving cognitive assessments and coping skills and combining practices to integrate learned techniques with real-life situations can reduce stress and anxiety and increase the quality of life [42]. In addition, mothers also benefit from group therapy; they feel like a part of a group with similar experiences and problems. It is more comfortable exchanging information and reducing worries by sharing each other’s experiences [43]. Different treatments such as GnRH analogues in central precocious puberty and tumor removal, blocking estrogen or androgen receptors, or treatment of hypothyroidism with Thyroid hormone replacement in peripheral precocious puberty are used to suppress symptoms of the disease [44]. All these processes cause psychological stress for both the patient and the parents. CBT approach can improve the treatment process and mental health along with the medications.

### Strength and limitations

Observing all the principles of clinical trials, including random allocation and allocation concealment, was among the strengths of this study. In order to reduce incorrect answers, the questionnaires were completed by the researcher and their native language was used to establish better communication with the participants in the counseling sessions. However, all the items answered by the participants were assumed to be correct because their validity determination was beyond the research methodology. The study was conducted among the mothers of elementary school students in Tabriz city. All of them were literate and had girls with precocious puberty symptoms, which can affect the generalizability of the results. It was uncertain whether mothers’ stress and anxiety were due to their daughters’ precocious puberty. Mental health was not evaluated in girls; it is suggested that this issue be investigated in future studies. Further research is needed for a more comprehensive conclusion.

## Conclusion

The precocious onset of puberty symptoms in girls may increase stress and anxiety levels in their mothers and result in a psychological burden on both. According to the results of this study, group CBT could decrease stress and anxiety and promote quality of life. However, more studies are required to make a definite conclusion in this field.

## Data Availability

The datasets generated and/or analyzed during the current study are not publicly available due to limitations of ethical approval involving the patient data and anonymity but are available from the corresponding author on reasonable request.

## Abbreviations

CBT: Cognitive-Behavioral Therapy
ANCOVA: Analysis of Covariance
95% CI: 95% confidence interval
IRCT: Iranian Registry of Clinical Trials
STAI: Spielberger State-Trait Anxiety Inventory
PSS: Perceived Stress Scale
SF-36: 36-item Short Form Health Survey
SD: standard deviation
